# Psychedelics and Other Psychoplastogens for Treating Mental Illness

**DOI:** 10.3389/fpsyt.2021.727117

**Published:** 2021-10-04

**Authors:** Maxemiliano V. Vargas, Retsina Meyer, Arabo A. Avanes, Mark Rus, David E. Olson

**Affiliations:** ^1^Neuroscience Graduate Program, University of California, Davis, Davis, CA, United States; ^2^Delix Therapeutics, Inc., Concord, MA, United States; ^3^Biochemistry, Molecular, Cellular, and Developmental Biology Graduate Program, University of California, Davis, Davis, CA, United States; ^4^Department of Chemistry, University of California, Davis, Davis, CA, United States; ^5^Department of Biochemistry and Molecular Medicine, School of Medicine, University of California, Sacramento, Sacramento, CA, United States; ^6^Center for Neuroscience, University of California, Davis, Davis, CA, United States

**Keywords:** psychoplastogen, ketamine, psilocybin, depression, neuroplasticity, prefrontal cortex, psychedelic, hallucinogenic

## Abstract

Psychedelics have inspired new hope for treating brain disorders, as they seem to be unlike any treatments currently available. Not only do they produce sustained therapeutic effects following a single administration, they also appear to have broad therapeutic potential, demonstrating efficacy for treating depression, post-traumatic stress disorder (PTSD), anxiety disorders, substance abuse disorder, and alcohol use disorder, among others. Psychedelics belong to a more general class of compounds known as psychoplastogens, which robustly promote structural and functional neural plasticity in key circuits relevant to brain health. Here we discuss the importance of structural plasticity in the treatment of neuropsychiatric diseases, as well as the evidence demonstrating that psychedelics are among the most effective chemical modulators of neural plasticity studied to date. Furthermore, we provide a theoretical framework with the potential to explain why psychedelic compounds produce long-lasting therapeutic effects across a wide range of brain disorders. Despite their promise as broadly efficacious neurotherapeutics, there are several issues associated with psychedelic-based medicines that drastically limit their clinical scalability. We discuss these challenges and how they might be overcome through the development of non-hallucinogenic psychoplastogens. The clinical use of psychedelics and other psychoplastogenic compounds marks a paradigm shift in neuropsychiatry toward therapeutic approaches relying on the selective modulation of neural circuits with small molecule drugs. Psychoplastogen research brings us one step closer to actually curing mental illness by rectifying the underlying pathophysiology of disorders like depression, moving beyond simply treating disease symptoms. However, determining how to most effectively deploy psychoplastogenic medicines at scale will be an important consideration as the field moves forward.

## Introduction

Theories regarding the etiology of depression and related neuropsychiatric diseases have evolved considerably in recent years. One of the oldest and most widely known theories posits that chemical imbalances in the brain are largely responsible for the development of neuropsychiatric diseases. Support for this theory originated with the observation in the 1950's that administration of the natural product reserpine induces depression (1). Reserpine depletes monoamine levels through inhibition of vesicular monoamine transporters (2), leading to subsequent degradation by monoamine oxidase (MAO) (3). Support for the chemical imbalance hypothesis (also known as the monoamine hypothesis) was further bolstered by findings that MAO inhibitors, tricyclics, and selective-serotonin reuptake inhibitors (SSRIs)—compounds that elevate synaptic levels of monoamines—all seemed to alleviate depressive symptoms (4). However, several pieces of evidence have emerged suggesting that the chemical imbalance hypothesis of depression is a drastic oversimplification.

**Figure 1:**
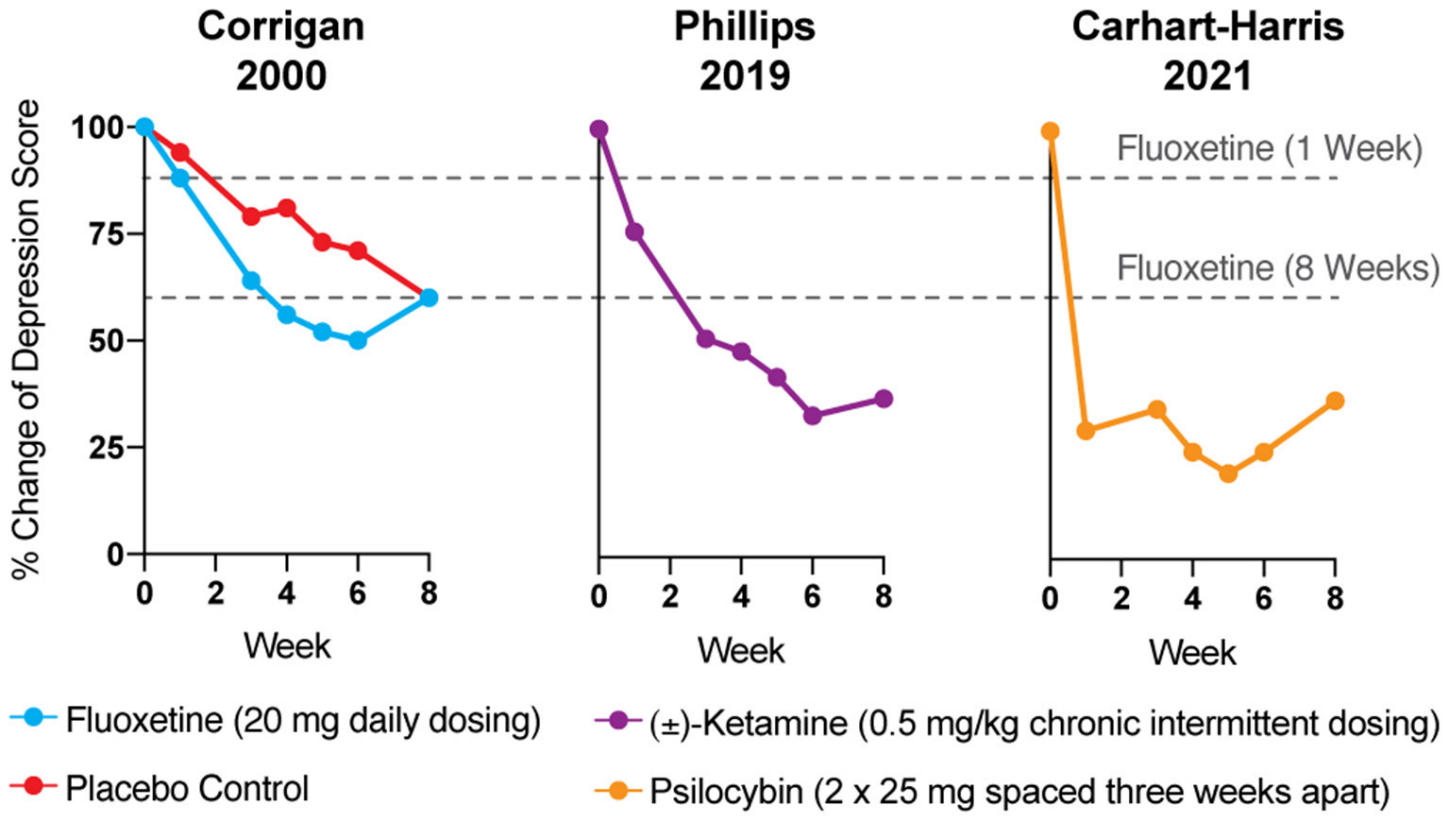
The antidepressant effects of psychoplastogens (e.g., ketamine and psilocybin) are more rapid and sustained than those of traditional antidepressants (e.g., fluoxetine). Data adapted from three clinical trials evaluating the effects of fluoxetine (6), ketamine (7), and psilocybin (8) for treating depression. Dashed lines represent the efficacy of either 1 or 8 weeks of fluoxetine treatment.

Reduction of monoamines through acute tryptophan or phenylalanine/tyrosine depletion does not induce depression in healthy subjects (5), which questions the causal role of “chemical imbalances” in depression. However, the primary issue with the monoamine hypothesis of depression lies in the temporal discrepancy between the acute effects of traditional antidepressants and their delayed therapeutic responses. Tricyclics, MAO inhibitors, and SSRIs all increase synaptic levels of monoamines in the brain within minutes, while it takes weeks before the antidepressant effects become apparent (Figure 1) (4, 9). In a patient population at heightened risk for suicide, the need for rapid-acting antidepressants is self-evident.

**Figure 2:**
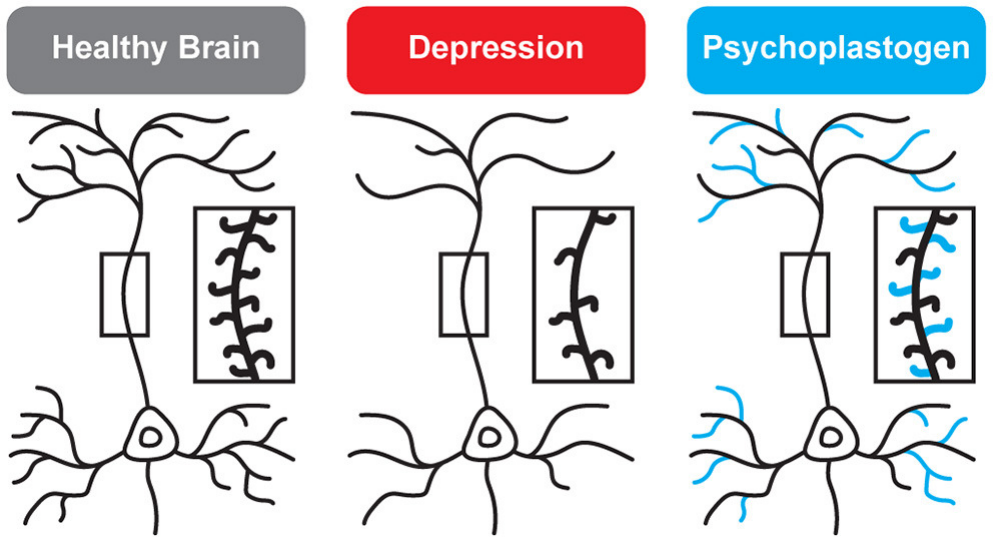
Genetic and environmental factors lead to the cortical atrophy observed in depression, which includes retraction of dendritic branches and loss of dendritic spines. These structural changes are reversed by psychoplastogens (12).

While traditional antidepressants acutely increase synaptic levels of monoamines, their chronic administration leads to changes in structural neuroplasticity that are contemporaneous with their clinical therapeutic effects (10–12). In fact, evidence suggests that chronic administration of traditional antidepressants can re-wire the brain, with chronic fluoxetine promoting ocular dominance plasticity in the visual cortex of adult rats (13). Such induced plasticity (iPlasticity) (14) has been hypothesized to play a major role in the actions of essentially all antidepressant treatments including slow-acting traditional antidepressants (15), transcranial magnetic stimulation (16), electroconvulsive therapy (15, 17), exercise (18), and acute sleep deprivation (19). Moreover, people (particularly males) with the brain-derived neurotrophic factor (BDNF) Val66Met single nucleotide polymorphism—a condition that reduces activity-dependent BDNF release (20)—are more likely to experience chronic depression (21, 22). These results lend substantial support to the neuroplasticity hypothesis of depression (sometimes referred to as the neurotrophin hypothesis) (11, 23–36). This hypothesis provides a strong conceptual framework for understanding mental illnesses as disorders of neural circuits induced by a combination of genetic and environmental factors (Figure 2). The corollary being that compounds capable of rectifying these circuit pathologies can potentially serve as a powerful, disease-modifying therapeutics.

Psychedelics—molecules with “mind-manifesting” properties—include pharmacologically diverse compounds such as dissociatives (e.g., ketamine), classic hallucinogens (e.g., LSD, psilocybin, DMT), and entactogens (e.g., MDMA). Several psychedelics have emerged as some of the most promising treatments for re-wiring pathological neural circuitry. Given their unusually robust abilities to produce rapid and long-lasting changes in neuronal structure and function following a single administration, these compounds have been classified as psychoplastogens—a term we coined to describe this new class of therapeutic compounds (37). Unlike traditional antidepressants, psychoplastogens produce both fast-acting and sustained beneficial behavioral effects after a single administration (Figure 1) (38–42). Here, we present evidence that directly targeting cortical circuits with psychoplastogens has the potential to produce enduring therapeutic responses in depression and co-morbid diseases. First-generation psychoplastogens are all hallucinogenic—they cause people to perceive things that are not real—which has important implications for how these medicines must be administered and how many patients could ultimately benefit from these treatment approaches. In this regard, non-hallucinogenic psychoplastogens offer significant advantages, with the potential to reach much larger patient populations and even replace traditional antidepressants as first-line treatments.

### Harnessing Structural Plasticity to Treat Mental Illness

Depression and related neuropsychiatric diseases are often viewed as stress-related disorders given the fact that they can be precipitated or exacerbated by chronic stress (43). In animals, chronic stress results in the prolonged release of glucocorticoids and leads to hypertrophy of the amygdala and nucleus accumbens, atrophy of the hippocampus and prefrontal cortex (PFC), and functional impairment of the PFC (44–55). Given the importance of the PFC in cognition and mediating top-down control over subcortical brain regions (56–66), these changes in neural circuitry are believed to underlie the deficits in learning/memory, mood, motivation, and reward seeking that are characteristic of depression and related disorders (10, 67–74).

Postmortem studies have demonstrated that patients with depression and related mental illnesses have lower *BDNF* and/or *TRKB* mRNA levels (75–77), reduced cortical neuron size (75, 78, 79), lower synaptic protein levels (80), decreased mTOR signaling (81), and fewer dendritic spines/synapses (82, 83) in the PFC. Clinical imaging studies have confirmed the results of these studies, demonstrating robust structural and functional deficits in the PFC across a range of disorders including depression, bipolar disorder, anxiety, obsessive compulsive disorder (OCD), schizophrenia, PTSD, alcohol abuse disorder, and substance abuse disorder (84–103). More recently, the advent of [^11^C]UCB-J (104) and [^18^F]UCB-J (105) has opened up new opportunities for using positron emission tomography (PET) imaging to measure the density of the synaptic protein SV2A *in vivo*. Using these new tools, depression severity was found to inversely correlate with SV2A density, and this neuronal atrophy was associated with aberrant network function as measured by magnetic resonance imaging (MRI) functional connectivity (106). Similar results were observed in patients with schizophrenia (107). Thus, substantial evidence points to the PFC as the convergence point underlying the pathophysiology of many neuropsychiatric diseases.

In imaging studies of traditional antidepressant treatment response, increased cortical thickness and cerebral blood flow in the PFC correlate with efficacy (108–112). Next-generation, psychoplastogenic antidepressants also modulate PFC function with both ketamine and psychedelics increasing PFC activation as measured by ^18^F-FDG PET imaging (113–116). In fact, antidepressant outcomes following ketamine treatment correlate well with PFC activation (117, 118). There is some evidence in humans that psychoplastogens can impact brain structure as well. Chronic use of the psychedelic tisane ayahuasca is associated with thickening of the anterior cingulate cortex (119), and ketamine treatment has been shown to rescue atrophy of the inferior frontal gyrus observed in MDD and PTSD patients (120).

Structural plasticity studies in preclinical animal models support the findings in humans suggesting that cortical neuron structure/function plays a key role in depression and related neuropsychiatric disorders. Cortical neuron atrophy and dysfunction is observed in rodents following chronic corticosterone administration (121), chronic unpredictable mild stress (122, 123), chronic restraint stress (48, 49, 124, 125), and chronic social defeat stress (126–128). These structural changes are accompanied by depressive phenotypes related to motivation (129), anxiety (122), and anhedonia (122, 129). Moreover, antidepressants appear to rectify these structural changes by promoting structural plasticity in the PFC.

Nature uses BDNF to induce structural plasticity in many neuronal populations, and direct administration of BDNF into the rodent brain has been shown to alleviate several depressive phenotypes, curb addiction, and enhance fear extinction (130–133). Conversely, disruption of BDNF signaling in the brain can block the behavioral effects of antidepressants. BDNF heterozygous mice are resistant to the effects of traditional antidepressants (134) and the psychoplastogen ketamine does not produce antidepressant-like effects in *BDNF* inducible knockout animals (135). The BDNF Val66Met single-nucleotide polymorphism causes major structural atrophy and functional deficits in the PFC and blocks the synaptogenic effects of ketamine (136, 137). Both rodents and humans with BDNF Val66Met polymorphisms exhibit impaired fear extinction learning and reduced mPFC activity during extinction (138), phenotypes that are common in stress-related disorders.

All antidepressant treatments impact BDNF/TrkB signaling in some way. Chronic, but not acute, administration of several traditional antidepressants has been shown to significantly increase mRNA levels of BDNF and/or TrkB as well as increase levels of cAMP response element binding protein (CREB), a transcription factor that regulates the expression of several proteins important for plasticity including BDNF (17, 139). Other studies have found that antidepressants from a variety of chemical families (e.g., SSRIs, SNRIs, tricyclics, etc.) all increase activation of TrkB and its downstream effector CREB (140). Moreover, the effects of the traditional antidepressant fluoxetine on synaptic plasticity and fear extinction have been shown to be dependent on BDNF (141, 142). Recent evidence suggests that several antidepressants, including traditional antidepressants, may directly interact with the TrkB receptor to facilitate TrkB signaling (143, 144). Taken together, the importance of BDNF/TrkB signaling in the therapeutic effects of traditional antidepressants is clear, even if these agents must be administered chronically to achieve robust regulation of this pathway.

Psychoplastogens are also known to impact BDNF/TrkB signaling, but in contrast to traditional antidepressants, they do so rapidly after a single administration. Ketamine increases BDNF protein translation (135, 145) and its antidepressant effects are absent when administered to inducible *BDNF* knockout mice (135) or homozygous mice harboring the BDNF Val66Met mutation (136). Ketamine and psychedelics modulate cortical neuron function by increasing dendritic spine and synapse density in the PFC (129, 146–149); however, ketamine's effects on structural plasticity appear to last for approximately a week (150) while psilocybin's effects seem to be more durable lasting for at least a month (151, 152). Though the primary molecular targets of ketamine and serotonergic psychedelics are distinct, their downstream pharmacology overlaps, requiring AMPA receptor, TrkB, and mTOR activation to elicit changes in neuronal structure and function (135–137, 148, 153, 154). Moreover, their effects seem to be C_max_ driven, as very short stimulation periods (15 min−1 h) are sufficient to induce sustained changes in cortical neuron structure (153). Psilocybin has also been shown to increase the density of SV2A *in vivo* as measured by PET imaging (155). Importantly, Liston et al. recently used a photoactivatable Rac1 to demonstrate that ketamine-induced spine growth in the PFC was causally related to long-lasting antidepressant effects of the drug in rodents (129). In humans, the subjective effects of ketamine wane after a few hours, but the antidepressant response continues to increase over several days (156). This time course is consistent with what we know about how ketamine and other psychoplastogens alter neuronal structure over time. An exceedingly short stimulation period (<1 h) is sufficient for psychoplastogens to activate cortical neuron growth mechanisms that can last for several days (153).

Several volatile anesthetics, such as isoflurane, nitrous oxide, propofol, and xenon, may produce rapid antidepressant effects (157–165). Xenon is not a small molecule, and by definition, is not a psychoplastogen. Other volatile anesthetics might be considered psychoplastogens if additional studies in larger patient populations confirm that they produce sustained therapeutic effects after a single administration. The data for isoflurane are encouraging as isoflurane produces rapid antidepressant effects after a single administration in both preclinical and in a subset of patients suffering from treatment-resistant depression (157–160). Moreover, these effects appear to be mediated *via* TrkB signaling and subsequent increases in dendritic spine density in the PFC and hippocampus (164, 165). Thus, preliminary evidence suggests that isoflurane may be considered a psychoplastogen.

### Possible Explanations for the Broad Therapeutic Potential of Psychoplastogens

**Figure 3:**
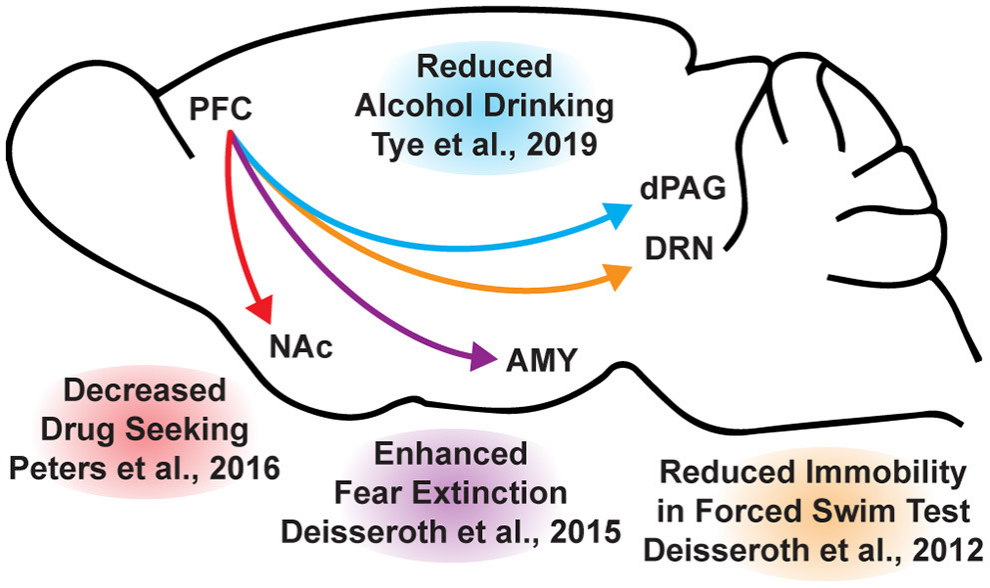
Opto- and chemogenetic experiments have revealed a number of circuits originating in the PFC that are relevant to the treatment of neuropsychiatric disorders. Arrows indicated excitatory projections.

While the beneficial effects of psychoplastogens can last for months following a single administration (40, 166, 167), and these medicines have demonstrated efficacy across a range of neuropsychiatric disorders including depression, PTSD, and addiction (156, 168–172), they are not panaceas. Their broad therapeutic utility likely arises from their ability to impact the structure/function of layer V pyramidal neurons in the PFC. As the PFC is a key hub impacted in most neuropsychiatric disorders, it is not surprising that psychoplastogens have proven useful for a variety of indications. Indeed, with the advent of opto- and chemogenetics, systems neurobiology has developed a much deeper understanding of what circuits control behavior. The PFC is known to exert top-down control over a variety of subcortical regions, and recent research has identified a number of circuits originating in the PFC that control behaviors relevant to the treatment of depression, anxiety, and addiction (Figure 3) (56, 173).

In mice subjected to social defeat stress, optogenetic stimulation of the ventral medial PFC increased social interaction and reduced anhedonia as measured *via* the sucrose preference test (174). Deisseroth et al. later found that optogenetic stimulation of medial PFC neurons projecting to the dorsal raphe nucleus (DRN) decreased immobility in the forced swim test (175). The forced swim test is a preclinical assay for antidepressant potential with high predictive validity (176, 177), and activation of this PFC → DRN circuit likely mediates the robust effects of psychoplastogens on forced swim test behavior. While the National Institutes of Mental Health (NIMH) has stressed that the FST is not a model of depression, it is a powerful behavioral readout for activation of the PFC → DRN circuit. The DRN is a serotonergic nucleus that has been implicated in major depressive disorder (178), and recent evidence suggests that acute activation of serotoninergic neurons in the DRN increase active coping to inescapable stress (179). Dzirasa et al. demonstrated that optical stimulation of layer V pyramidal neurons in the PFC expressing channelrhodopsin-2 was sufficient to produce an antidepressant-like response in the forced swim test and suppress anxiety-like behavior in the elevated plus maze for over 10 days after the last optical stimulation session (180). This type of long-lasting anxiolytic effect is reminiscent of sustained effects observed following a single administration of a psychoplastogen. Finally, Duman et al. found that optogenetic stimulation of the infralimbic cortex produces rapid and sustained antidepressant-like effects comparable to ketamine in the forced swim, novelty-suppressed feeding, and sucrose preference tests (181). Moreover, microinfusion of ketamine into the infralimbic cortex produces comparable antidepressant-effects as systemic administration of ketamine and inactivation of the infralimbic cortex with muscimol was sufficient to block the antidepressant-like effects of systemic ketamine (181).

In addition to relieving symptoms associated with depression, psychoplastogens have also demonstrated efficacy for treating PTSD—a disorder that can involve dysfunction of the amygdala (AMY). The medial PFC and amygdala are well-connected, and bi-directional communication between these structures is likely involved in modulating responses to emotional stimuli (182). In fact, optogenetic stimulation of ventral medial PFC projections to the basomedial amygdala decreases fear responses and facilitates fear extinction learning (183). Chemogenetic inhibition of PFC neurons projecting to the amygdala is sufficient to impair fear extinction learning (184). As PTSD has often been described as a disorder of impaired fear extinction (73, 185–187), the therapeutic effects of psychoplastogens might result from their ability to strengthen PFC → AMY circuits mediating top-down control of fear responses.

Like fear extinction, drug-cue extinction is believed to involve neurons in the PFC (59). This is perhaps unsurprising given the large body of neuroimaging data suggesting that PFC hypofunction is a hallmark of addiction (68). Chemogenetic activation of ventral medial PFC neurons projecting to the nucleus accumbens (NAc) shell was able to reduce cue-induced reinstatement of drug-seeking behavior (188). Moreover, chronic cocaine self-administration has been shown to decrease the intrinsic excitability of pyramidal neurons in the PFC (189). Optogenetic stimulation of these neurons prevented compulsive drug-seeking while silencing these neurons promoted drug-seeking behavior despite being paired with aversive foot shocks (189). Similarly, pharmacological activation and inactivation of neurons in the infralimbic cortex suppressed and enhanced reinstatement of drug-seeking behavior, respectively (190). Optogenetic experiments have also revealed that ventral medial PFC projections to the NAc shell are involved in suppressing ethanol self-administration in the presence of aversive stimuli (191). A PFC → NAc circuit appears to be involved in compulsive food-seeking behavior as well, given that chemogenetic inhibition of this circuit led to compulsive food seeking even in the presence of aversive foot shocks (192). While PFC → NAc circuits have been well-established in controlling drug-seeking behavior, more recently, Tye et al. demonstrated that a PFC projection to the dorsal periaqueductal gray (dPAG) may also play an important role in addiction (193). Specifically, they showed that optogenetic activation of a PFC → dPAG circuit prevented compulsive alcohol consumption.

Psychoplastogens produce robust, fast-acting, and long-lasting effects on structural plasticity in the PFC. This may explain why they have demonstrated efficacy in many preclinical rodent behavioral tests involving PFC circuitry including the forced swim test and fear extinction learning (151, 194–197). However, achieving circuit-level selectivity is a key challenge in the design of optimized psychoplastogens with minimal to no side effects. The issue with non-selective activation of BDNF/TrkB signaling is apparent from chronic stress studies demonstrating that enhanced BDNF/TrkB signaling in the amygdala leads to maladaptive plasticity resulting in overactivation of this brain region and exacerbated anxiety and fear responses (47, 198–200). Furthermore, compounds that promote plasticity in the mesolimbic pathway could have pro-depressive and/or addictive properties (201, 202).

Psychoplastogens that target the 5-HT2A receptor have advantages over NMDA receptor antagonists like ketamine, as 5-HT2A receptors exhibit a relatively selective expression profile. With the exception of the claustrum, the highest density of 5-HT2A receptors is in layer V pyramidal neurons of the PFC, which are precisely the neurons that are most impacted in stress-related neuropsychiatric diseases. In rodents, this expression pattern has been confirmed using immunohistochemistry (203–206), light and electron microscope immunocytochemistry (203), *in situ* hybridization (207, 208), receptor autoradiography (209), and transgenic mice expressing EGFP under control of the 5-HT2A receptor promoter (210). A similar pattern of 5-HT2A receptor expression has been shown in human post-mortem tissue using both autoradiography (211) and *in situ* hybridization (212). Additionally, PET imaging has revealed a high density of 5-HT2 receptors in the frontal and temporal cortices of the human brain (213). The high genetic localization of 5-HT2A receptors to excitatory neurons in layer V of the PFC is perhaps why animals do not typically self-administer classic serotonergic psychedelics (214, 215) and most psychedelics are not considered to be addictive (216, 217).

### Can the Intoxicating Effects of Psychedelics Be Removed to Create More Scalable Therapeutics?

At high doses, psychedelics reliably induce both hallucinations and mystical-type experiences. Currently, it is unclear if the mystical-type experiences they induce are necessary for their therapeutic effects in humans (218, 219). Moreover, it is unclear if the intoxicating effects of psychedelics can be decoupled from their therapeutic properties. This critical question has profound implications for healthcare, as hallucinogenic treatments will inevitably be more limited in scope given safety and cost considerations. Many patients describe psychedelic-induced “peak” or “mystical” experiences as being among the most meaningful events of their lives, and the intensity of these events correlates with therapeutic responses (40, 220–226). While these events could provide patients with valuable insight relevant to their disease symptoms, it is important to remember that correlation does not imply causation, and mystical-type experiences could simply be an epiphenomenon associated with 5-HT2A receptor activation (227, 228). Activation of 5-HT2A receptors also promotes structural and functional neuroplasticity (148), which could be the primary driver of the sustained behavioral effects following a single administration of ketamine or serotonergic psychedelics.

Given that 5-HT2A receptor activation is associated with both psychoplastogenic effects (148) and mystical-type experiences (227, 228), it is challenging to determine exactly how much each contributes to the therapeutic properties of psychedelics. However, a number of key pieces of evidence suggest that intoxicating subjective effects are *not necessary* to achieve some level of therapeutic efficacy. In patients treated with ketamine, floating sensations did not correlate with PFC activation as measured by ^18^F-PET, demonstrating that it is possible to activate cortical circuits to produce antidepressant responses without inducing dissociative effects (117). In fact, several studies have demonstrated that intraoperative ketamine administration can improve postoperative mood even though the patients were unconscious at the time of administration (229–231). This strongly suggests that the dissociative experience itself is not necessary for ketamine to produce antidepressant effects.

Additionally, there is preclinical evidence suggesting that *R*-ketamine is a potent psychoplastogen that induces longer-lasting antidepressant-like effects than the S-enantiomer (i.e., Spravato^®^) despite having a lower affinity for the NMDA receptor and producing fewer dissociative effects (232, 233). Similarly, a metabolite of *R*-ketamine lacking dissociative properties has also demonstrated robust antidepressant-like effects in rodents (234). Future clinical trials using these non-hallucinogenic agents will prove informative when assessing the role of subjective effects in ketamine treatment response.

While *R*-ketamine is still in the early stages of clinical development, 3,4-methylenedioxymethamphetamine (MDMA) has demonstrated robust results in a recently disclosed Phase III trial (235). As an atypical psychedelic of the entactogen family (236), MDMA produces robust effects on cortical neuron growth (148), and facilitates fear extinction learning (197) through a 5-HT2-dependent mechanism (237) without inducing psilocybin- or ketamine-like perceptual effects or dissociation. In fact, only about 20% of recreational MDMA users report experiencing any visual hallucinations, and these are relatively mild compared to those induced by psilocybin and LSD (236, 238, 239). While MDMA does induce a “blissful state,” likely due to its effects on monoamine efflux, it does not produce mystical-type experiences as measured using a variety of scales related to altered states of consciousness (240, 241). This is in stark contrast to drugs like ketamine and psilocybin.

As an entactogen, MDMA is pharmacologically distinct from classic serotonergic psychedelics like psilocybin and LSD. However, there is clinical evidence suggesting that non-hallucinogenic analogs of classic psychedelics can also produce therapeutic effects. Lisuride, a non-hallucinogenic structural analog of LSD, has been shown to have an antidepressant properties in the clinic (242). Moreover, lisuride has demonstrated some efficacy in preclinical models as well (243). Lisuride has a polypharmacology profile that includes activation of D2 and 5-HT1A receptors in addition to 5-HT2 receptors, and thus, it is currently unclear what receptor(s) mediate its antidepressant effects.

In addition to the clinical evidence supporting the efficacy of non-hallucinogenic psychoplastogens, mounting preclinical data suggest that the beneficial effects of psychoplastogens can be dissociated from their hallucinogenic effects. In 2019, we demonstrated that low doses of DMT produce beneficial effects in rodent behavioral paradigms relevant to treating depression and PTSD comparable to high doses (194, 195). The low dose was predicted to be subhallucinogenic in humans based on allometric scaling while the high dose was predicted to be hallucinogenic. However, the data on human psychedelic microdosing are equivocal, and therapeutic benefit of low doses of psychedelics remains to be demonstrated in a well-controlled clinical study (244–249). Moreover, administration of low doses of hallucinogenic compounds is not an ideal therapeutic strategy, as these compounds still possess the potential for abuse. Fortunately, through careful chemical design, we were able to engineer several *non-hallucinogenic* analogs of psychedelics with beneficial properties (250–252). These compounds do not induce a head-twitch response—a behavioral proxy for hallucinations in mice that correlates exceptionally well with hallucinogenic potency in humans (253, 254).

Our two most advanced compounds—AAZ and TBG—have demonstrated robust plasticity-promoting properties and produce sustained (>1 week) antidepressant-like effects following a single administration in both environmental (chronic unpredictable stress) and genetic (VMAT2 heterozygous mice) models of depression as measured *via* behavioral tests relevant to motivation, anhedonia, anxiety, and cognitive flexibility (250, 251, 255). TBG has also been shown to have anti-addictive properties in models of alcohol and opioid use disorders (250). Moreover, a single dose of TBG was able to completely rescue circuit-level dysfunction induced by chronic stress, which included deficits in dendritic spine density, calcium dynamics, and interneuron function (255). The psychoplastogenic effects of TBG and AAZ may involve activation of 5-HT2 receptors (250, 251, 256), though detailed mechanistic studies have not yet been reported.

One of the most important questions to address is the durability of psychoplastogen effects. In both humans and rodents, the antidepressant responses of ketamine appear to last for about 1 week. This correlates well with the effects of the drug on dendritic spine density (129, 150). In contrast, the mood-elevating properties of psilocybin seem to last significantly longer (40–42, 151), as do its effects on neuronal structure (152). Currently, it is unclear exactly how long the effects of non-hallucinogenic psychoplastogens will last following a single administration, and head-to-head comparisons with ketamine and psilocybin are warranted to help establish optimal dosing frequency.

### Patient Populations Best Suited for Hallucinogenic and Non-hallucinogenic Approaches

Given that ~20% of American adults suffer from a mental illness in a given year (256), the economic burden of these disorders is estimated to be in the hundreds of billions of dollars annually (257, 258). Moreover, the current standard of care treatments suffer from slow onset (e.g., 7 week average response time with citalopram) (259), low efficacy (only 27% of depressed patients achieved remission after 12 weeks of citalopram treatment as measured by HAM-D) (259), and often intolerable side-effects (260), it is clear that we need to expand psychiatry's arsenal to include a variety of new approaches. Both hallucinogenic and non-hallucinogenic psychoplastogens have important roles to play in the fight against mental illness, but it is critical to identify which patient populations are best served by these types of treatment. First, many patients may not want to participate in psychedelic-assisted therapy (261) given that hallucinogens can induce acute anxiety and may lead to challenging experiences (40). For those who are open to psychedelic-assisted therapy, significant barriers to treatment still exist.

Currently, there are over 300 clinical trials registered on clinicaltrials.gov^1^ to study the effects of psychedelics in humans with the majority of recent studies focusing on the effects of psilocybin and MDMA (262, 263). However, only a fraction of patients who volunteer for psychedelic-assisted therapy are permitted to participate. Exclusion criteria for these trials typically include cardiovascular and mental health risks that could potentially be exacerbated by psychedelics. For example, psychedelic-assisted therapy is generally contraindicated for people with a family history of psychotic disorders or complex psychiatric comorbidities to avoid the possibility of triggering a first episode of psychosis or precipitating suicidal behaviors (264).

**Figure 4:**
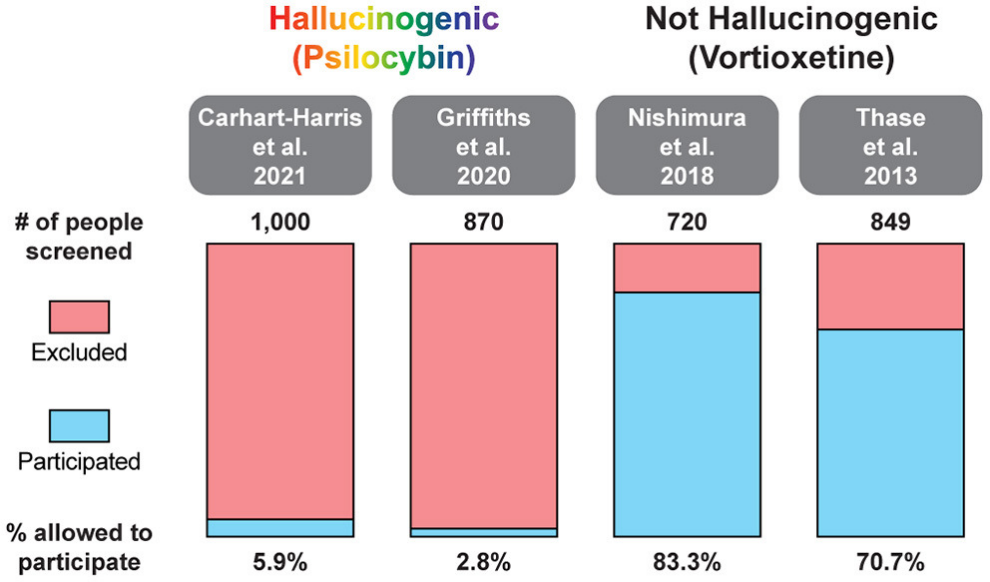
Percentage of patients excluded from recent large MDD trials of the hallucinogenic drug psilocybin and the non-hallucinogenic drug vortioxetine. A comprehensive analysis of all psychedelic trials is beyond the scope of this review. For additional information on the broad range of smaller trials with psychedelics, please see (262, 263).

**Figure 5:**
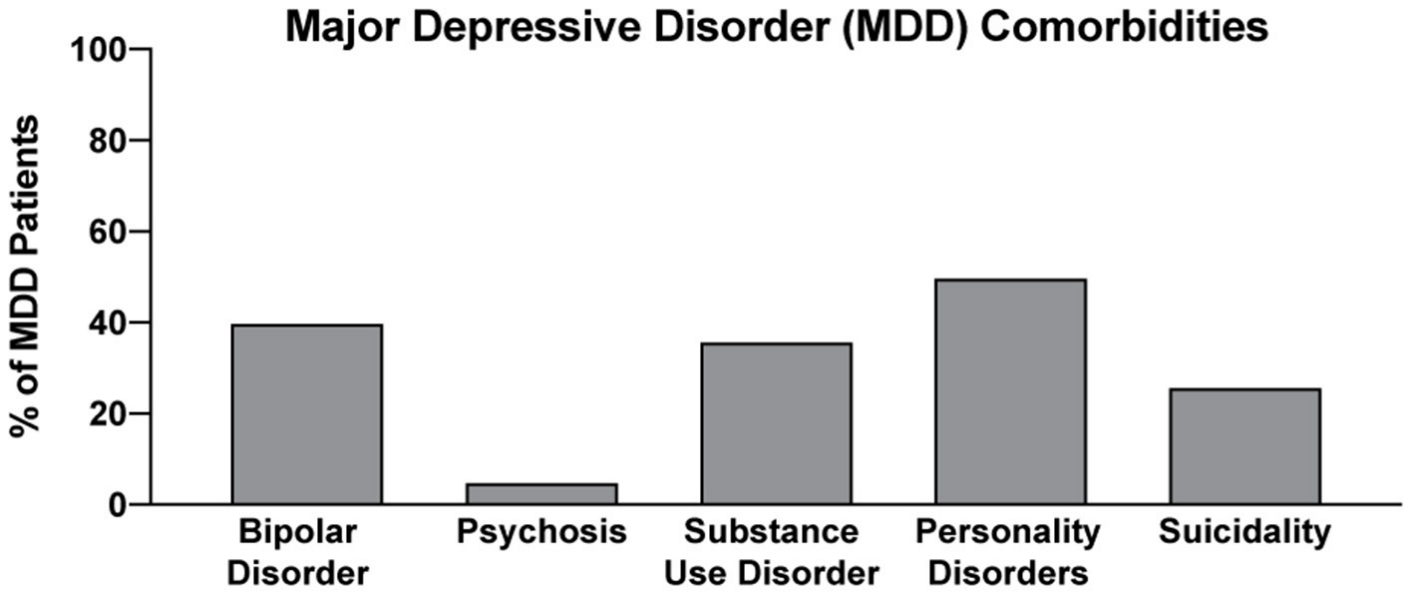
Percentage of MDD with various comorbidities. Data for bipolar disorder, psychosis, substance use disorder, personality disorders, and suicidality were obtained from references (267–271), respectively.

In two recent psilocybin clinical trials, ~95% of all volunteers were eliminated on the basis of exclusion criteria (Figure 4) (8, 42). For comparison, ~25% of participants were excluded from two recent major depressive disorder (MDD) trials of the non-hallucinogenic compound vortioxetine (Figure 4) (265, 266). If these strict exclusion criteria are deemed necessary by the FDA and payers, they will drastically limit the number of patients who could potentially benefit from this treatment paradigm, especially when you consider the high genetic heritability and co-morbidity of neuropsychiatric disorders (Figure 5) (272–278). Between 33 and 66% of patients suffering from MDD have a psychiatric comorbidity that could potentially exclude them from psychedelic-assisted therapy (267–271, 279, 280).

Identifying factors that can predict how patients will respond to psychedelic-assisted therapy will be important for maximizing efficacy and reducing adverse events. For example, patients who exhibit apprehension, preoccupation, or baseline attachment avoidance appear to be more likely to have challenging experiences following administration of a psychedelic drug (281, 282). Using these metrics to pre-screen patients should improve the safety and efficacy of psychedelic-assisted therapy; however, stratification of patients in this manner will inevitably reduce the addressable patient population, again, emphasizing the need for a non-hallucinogenic first-line treatment against depression and related disorders.

Presumably, treatment with non-hallucinogenic psychoplastogens would not be limited by comorbidities or other factors that might exclude someone from participating in psychedelic-assisted therapy. For these reasons, and others described below, it seems reasonable for non-hallucinogenic psychoplastogens to potentically be used as first-line treatments, assuming that they demonstrate greater efficacy in humans than the standard of care (i.e., traditional antidepressants). Psychedelic-assisted therapy could be reserved for patients who have not responded to any other medicine. Indeed, some patients may benefit from the mystical-type experiences occasioned by psychedelics as many people rate these experiences as among the most meaningful in their lives. Such a positive experience could have a variety of effects on patients including, but not limited to, improving the relationship between therapist and patient, helping patients to gain insight about their condition, or producing a powerful placebo effect. It is challenging to design truly double-blind placebo-controlled clinical trials with psychedelics given their profound subjective effects. Despite efforts to employ “active placebos” (e.g., niacin or a low-dose of a psychedelic) (40, 220), many patients and clinicians can still correctly distinguish between a high dose of a psychedelic drug and an active placebo. Thus, the exact role of placebo effects in the overall efficacy of psychedelic-assisted therapy has not been firmly established. Even so, an enhanced placebo effect can potentially be leveraged to treat severely depressed patients who have not been helped by other means (283–285).

### Healthcare System Issues With the Psychedelic-Assisted Psychotherapy Model

In addition to comorbidities and genetic predispositions, the basic mechanics of healthcare systems are likely to be major factors limiting the number of patients who will ultimately be able to receive hallucinogen-based therapeutics. Given their profound effects on perception, these drugs necessitate administration in a clinical setting where the patient can be observed by medical professionals. In 2019, Johnson & Johnson's Spravato^Ⓡ^ (esketamine nasal spray) became the first hallucinogenic psychoplastogen treatment approved by the FDA for refractory depression (286), and in 2020 the indication was expanded to include adult major depressive disorder (287). Spravato^Ⓡ^ has to be administered in a clinic under supervision due to the known risks of serious adverse outcomes resulting from disassociation and sedation as well as the potential for abuse. After intake and medical screening, the patient is enrolled in the Spravato^Ⓡ^ REMS (Risk Evaluation and Mitigation Strategy) program. Spravato^Ⓡ^ is self-administered intranasally in the presence of a healthcare professional, and the patient is monitored for the next 2 h. Patients receive two treatments a week for the 1st month, and once a week or once every 2 weeks after that. For racemic ketamine administered intravenously, clinics follow the NIMH trial protocol of six infusions administered over a two-to-3-week period in an outpatient clinic or medical facility. Boosters are given every 3–5 weeks after that. Intravenous infusion takes about 40 min, and guidance is provided not to drive, operate any dangerous machinery, or make any important decisions until the day after a ketamine treatment. The requirement for administration in a clinical setting drastically increases the cost of both racemic ketamine and Spravato^Ⓡ^.

Though psychedelics are expected to be administered less frequently than ketamine given their robust effects, they too must be administered under the care of a healthcare professional. Current research-based psychedelic-assisted therapy has three phases: preparation, treatment, and integration. Given that psychedelics have the potential to cause dangerous behaviors due to potential negative psychological reactions such as anxiety, fear, panic, or psychosis, a team of two professional therapists is required to be in attendance to supervise, but minimally interact with the patients throughout the course of the drug's action (288). Moreover, it is recommended that multiple healthcare workers be involved in all three stages to ensure that professional boundaries are maintained (289). The preparation session establishes the alliance between the therapist and the participant. Treatment sessions, which typically last 6–8 h for psilocybin-assisted therapy (288) allow the participants to have a peak experience within a set and setting thought to be most conducive to optimizing the therapeutic effect. The integration session is meant to help the participant process, rationalize, and gain insight from the hallucinogenic experience. Although psychedelic therapies have demonstrated outstanding benefits in several clinical trials (290), the cost-effectiveness and overall accessibility of such therapies raises major concerns.

**Figure 6:**
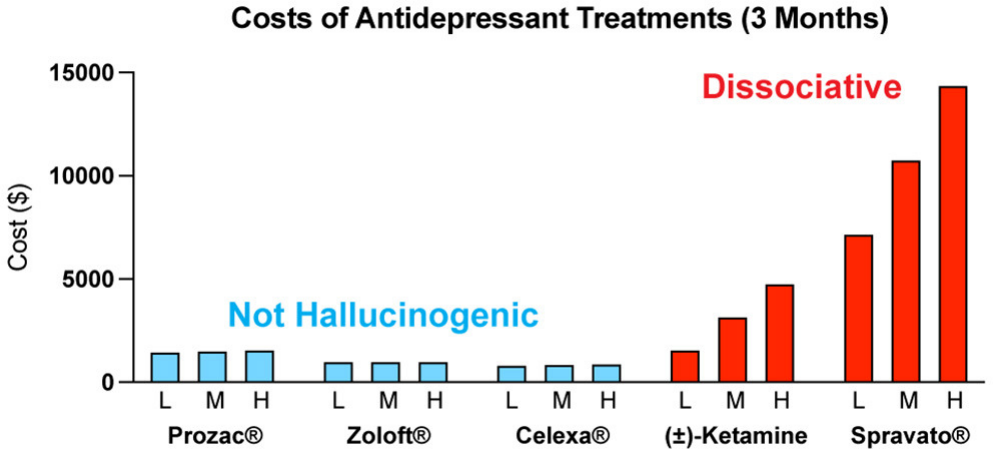
Estimated quarterly (3 months) costs (L = low, M = medium, H = high) for daily administration of non-hallucinogenic take-home traditional antidepressants (blue) and intermittent dosing of clinically administered, dissociative psychoplastogen (ketamine) treatments (red). Note: generic ketamine costs include drug, administration (IV), and required monitoring, while Prozac^®^, Zoloft^®^, Celexa^®^, and Spravato^®^ costs are for the drug only. Retail costs for SSRIs reflect the low and high branded price published on goodrx.com^2^ with the medium price being calculated as an average of the two. To estimate the total cost, we used medium doses for each drug (Prozac^®^ 20 mg, Zoloft^®^ 50 mg, and Celexa^®^ 20 mg,) and assumed daily dosing for 90 days. Ketamine infusion cost was sourced from ketamineclinicsdirectory.com/ketamine-infusion-cost/.^3^ This site estimates the low and high cost of a complete set of 4–6 generic ketamine infusions to be $1,600–$4,800, respectively. The medium cost was calculated as an average of the two ($3,200). Our estimate for the 3-month cost of generic ketamine assumes that no additional doses are needed beyond the initial 4–6 doses. Retail Spravato^®^ costs were estimated based on the price of a 56 mg dose ($900) published on goodrx.com^3^ and dosing was based on the package insert (Revised 7/2020). The label indicates Spravato^®^ should be dosed twice a week during the induction phase (weeks 1–4; $900 × 2 × 4 = $7,200), once a week during the maintenance phase (weeks 5–8; $900 × 1 × 4 = $3,600), and once a week thereafter (weeks 9–12; $900 × 1 × 4 = $3,600). The estimated low cost of Spravato^®^ includes the induction phase only. The estimated medium cost of Spravato^®^ includes the induction phase and the maintenance phase. The estimated high cost of Spravato^®^ includes the induction phase, maintenance phase, and 4 weeks of additional treatment. Note: the cost of administration and monitoring for Spravato are not publicly available, and thus not included, but these costs are anticipated to be significant.

The costs associated with treating mental illness with hallucinogenic psychoplastogens is extremely high compared to the standard of care (Figure 6). For example, the initial month of ketamine therapy costs from $4,720 to $6,785, and subsequent monthly therapy can range from $2,360 to $3,540 (291). Additional costs associated with patients taking time off work to receive treatment and to travel to appropriately staffed/equipped clinics must also be considered.

Many people assume that psilocybin-assisted therapy will be cheaper than ketamine treatment as the antidepressant effects following a single administration of psilocybin appear to be more enduring than those of ketamine (292). However, the overall cost of psilocybin treatment is estimated to be close to, if not higher than, the cost of Spravato^Ⓡ^ treatment due to the increased session participation of therapists. The low-throughput nature and associated high costs of the psychedelic-assisted therapy model have been acknowledged by the community and have resulted in new studies being launched by non-profit and corporate sponsors to streamline the process and reduce costs through group and virtual therapy sessions (293). However, it is currently unclear if such approaches will be as safe and efficacious as the current model.

The cost associated with rolling out the psychedelic-assisted therapy model poses a great hurdle to its effective implementation in the current mental health ecosystem. In 2019, the Institute for Clinical and Economic Review (ICER) issued a recommendation that deemed Spravato^Ⓡ^ to deliver a “low value for money” according to their value-assessment framework (294). Furthermore, the UK price watchdog agency, the National^2^,^3^ Institute for Health and Care Excellence (NICE), refused to endorse Spravato^Ⓡ^ therapy for inclusion as a reimbursable drug on the UK's National Health System (NHS) (295). Although NICE acknowledged the drug's efficacy for relieving the symptoms of depression, the agency also commented that the “introduction of esketamine into clinical practice in the NHS will be complex because the structure and delivery of services would need to be changed. Estimates of the costs of providing the clinical service for esketamine were highly uncertain.” As these price watchdog agencies become progressively more influential in the decision-making process of payers, their recommendations will likely lead to payers raising their medical criteria for coverage, potentially jeopardizing patient access to psychedelic-assisted therapy for financial reasons. We are already seeing racemic ketamine being administered off-label for a variety of neuropsychiatric disorders without reimbursement from insurance companies (296). This leads to an inequity issue, where only wealthy individuals who can afford the out-of-pocket costs have access to this type of treatment.

In addition to the financial burdens to the patients, understaffing of qualified psychotherapy practitioners is likely to be one of the biggest issues for nationwide implementation of psychedelic-assisted therapy. The FDA requires all U.S. therapists to have at least a master's degree, and current best practices require a minimum of two therapists to be present during psychedelic sessions (288). Moreover, all the therapists who participate in the psilocybin and MDMA clinical development programs are required to hold a professional license and demonstrate clinical experience in psychotherapy or mental health counseling (288). Although institutes such as the California Institute of Integral Study (CIIS), Multidisciplinary Association for Psychedelic Studies (MAPS), and corporate programs from companies like COMPASS Pathways offer short-term training programs for psychedelic-assisted counseling, there is a huge demand and supply gap for competent therapists considering that the estimated prevalence of treatment-resistant depression (TRD) in the U.S. is around 2.8 million people (258). Moreover, given that set and setting are well-known to influence the subjective effects of psychedelics (288, 297), clinical centers with appropriate facilities will need to be established.

### Democratizing Access to Psychoplastogenic Medicines

The limitations associated with hallucinogenic medicines could prevent many patients from benefiting from the growing body of psychedelic-inspired research related to pathological circuit remodeling. By eliminating the need to treat patients in the clinic, non-hallucinogenic psychoplastogens—which would presumably be available from retail pharmacies much like traditional antidepressants—would reduce the complexity of treatment administration and have the potential to greatly expand access of patients to psychoplastogenic medicines. These molecules produce the same types of long-lasting structural and functional changes in the brains of preclinical animals that follow administration of ketamine or serotonergic psychedelics. Assuming that they are efficacious in humans, non-hallucinogenic psychoplastogens would not bear the same administrative costs and limitations as first-generation hallucinogenic psychoplastogens. When comparing the known or predicted yearly healthcare costs associated with ketamine, psilocybin, SSRIs, and non-hallucinogenic psychoplastogen treatments, it is clear that the non-hallucinogenic approaches will be more cost-effective (Figure 6).

Non-hallucinogenic psychoplastogens could potentially meet the demands of patients who fail to respond to monoaminergic agents, or even replace them as more effective first-line treatments with fewer side-effects. In preclinical animal models, like in humans, traditional monoaminergic antidepressants require chronic administration to achieve robust efficacy (298). Such chronic dosing paradigms inevitably lead to a host of undesirable side-effects that include weight gain, sexual dysfunction, and gastrointestinal problems (299), with many patients refusing to take traditional antidepressants due to their side-effects (260). In contrast, non-hallucinogenic psychoplastogens, like their hallucinogenic counterparts, produce sustained therapeutic behavioral responses in preclinical animal models after a single administration (250, 251). Thus, the need for chronic dosing in humans will likely be obviated resulting in fewer undesired side-effects. However, a major challenge for the field will be to determine exactly what frequency of dosing will be most effective. Fortunately, new imaging tools have the potential to identify biomarkers of psychoplastogen efficacy. These include relatively new positron emission tomography (PET) tracers (104, 105) that can non-invasively measure the effects of psychoplastogens on synaptic vesicle density *in vivo* (155).

**Figure 7:**
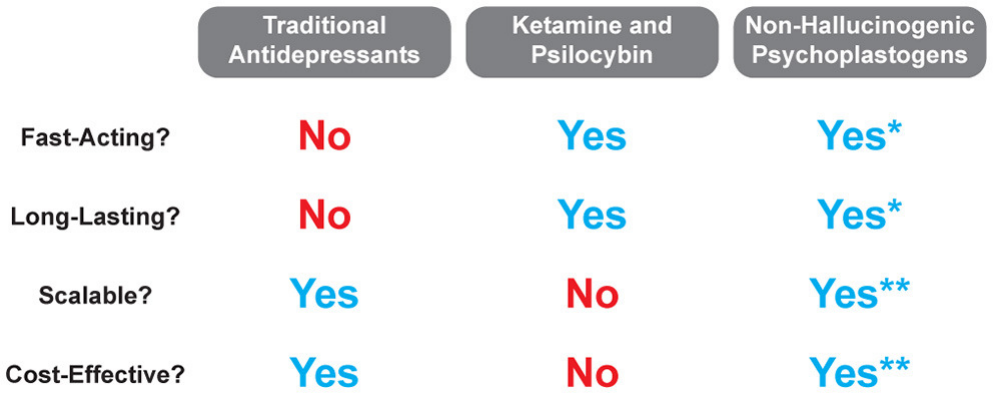
Comparison of traditional antidepressant treatments with hallucinogenic and non-hallucinogenic psychoplastogen treatments. *Currently, no clinical trials have been conducted with non-hallucinogenic psychoplastogens, and thus, their fast-acting and long-lasting effects refer to preclinical testing only. **The scalability and cost-effectiveness of non-hallucinogenic psychoplastogens are based on the assumption that they will produce clinical efficacy greater than the standard of care (i.e., traditional antidepressants).

It is quite possible that insight gained from mystical-type experiences coupled with changes in neurocircuitry is responsible for the large effect size and durability of psychedelic-assisted therapy. However, if even a fraction of the efficacy or durability could be achieved using compounds that do not induce mystical-type experiences or hallucinations, a much larger patient population could benefit. While mystical-type experiences will undoubtedly be beneficial for some patients, they may not be necessary for all patients. Thus, if non-hallucinogenic psychoplastogens can demonstrate efficacy in the clinic as robust as their effects in preclinical models, their advantages over both traditional monoaminergic antidepressants and hallucinogenic psychoplastogens should position them as first-line treatment options (Figure 7).

## Conclusion

The ability to selectively modulate neural circuits using small molecule psychoplastogens opens up new horizons in neuropsychiatry focused on healing pathological neural circuitry rather than masking disease symptoms. This type of circuit-based approach represents a fundamental shift in how we might treat a number of neuropsychiatric diseases and has important implications for the future of CNS drug discovery. Given the history of neuropsychiatry and the intractable nature of brain disorders, we need to take advantage of every available tool in our therapeutic arsenal including both hallucinogenic and non-hallucinogenic psychoplastogens. Ketamine and psilocybin have demonstrated that it is possible to produce long-lasting beneficial changes in neural circuitry using small molecule drugs, and they have forged a path for future, optimized psychoplastogens to take their place. If we ever hope to heal the nearly 20% of the population suffering from a mental illness, we must find innovative ways to reduce healthcare costs and broaden patient access to psychoplastogenic medicines. Non-hallucinogenic psychoplastogens have the potential to be truly scalable solutions to many of the problems facing neuropsychiatry.

## Author Contributions

DO wrote the manuscript with input from all authors. MV and AA made significant contributions to the introduction and the sections titled Harnessing Structural Plasticity to Treat Mental Illness and Possible Explanations for the Broad Therapeutic Potential of Psychoplastogens. RM and MR made significant contributions to the sections titled Patient Populations Best Suited for Hallucinogenic and Non-Hallucinogenic Approaches, Healthcare System Issues with the Psychedelic-Assisted Psychotherapy Model, and Democratizing Access to Psychoplastogenic Medicines. All authors contributed to the article and approved the submitted version.

## Funding

This work was supported by funds from the National Institutes of Health (NIH) (R01GM128997 to DO and 5T32GM099608 to MV). The authors declare that this study received funding from Delix Therapeutics Inc. The funder was not involved in the study design, collection, analysis, interpretation of data, the writing of this article or the decision to submit it for publication.

## Conflict of Interest

DO is a co-founder and chief scientific officer of Delix Therapeutics, Inc. MR and RM are employees of Delix Therapeutics. The remaining authors declare that the research was conducted in the absence of any commercial or financial relationships that could be construed as a potential conflict of interest.

## Publisher's Note

All claims expressed in this article are solely those of the authors and do not necessarily represent those of their affiliated organizations, or those of the publisher, the editors and the reviewers. Any product that may be evaluated in this article, or claim that may be made by its manufacturer, is not guaranteed or endorsed by the publisher.

## Footnotes

^1^Clinical trials. https://clinicaltrials.gov/ [Accessed June 12, 2021]. (2021). Keyword: psychedelic including synonyms hallucinogen and hallucinogenic drugs. We identified 316 studies. Re-querying the database in August 2021 we identified 324 studies of which 151 studies are completed.

^2^Data were obtained from goodrx.com [accessed June 2021].

^3^Data for racemic ketamine were obtained from https://ketamineclinicsdirectory.com/ [accessed June 2021].
